# Killing of Kaposi's sarcoma-associated herpesvirus-infected fibroblasts during latent infection by activated natural killer cells

**DOI:** 10.1002/eji.201040661

**Published:** 2011-05-27

**Authors:** Nick C Matthews, Martin R Goodier, Rebecca C Robey, Mark Bower, Frances M Gotch

**Affiliations:** 1 Department of Immunology, Chelsea and Westminster Hospital, Imperial College LondonLondon, UK; 2 Department of Oncology, Chelsea and Westminster Hospital, Imperial College LondonLondon, UK

**Keywords:** Immune evasion, Kaposi's sarcoma-associated herpesvirus, NK cells

## Abstract

Kaposi's sarcoma-associated herpesvirus (KSHV) establishes life-long infection by evading clearance by the host immune system. In de novo infection and lytic replication, KSHV escapes cytotoxic T cells and NK cells through downregulation of MHC class-I and ICAM-1 molecules and associated antigens involved in forming and sustaining the immunological synapse. However, the efficacy of such mechanisms in the context of the predominantly latent KSHV infection reported in Kaposi's sarcoma (KS) lesions is unclear. Using primary dermal fibroblasts in a novel in vitro model of chronic latent KSHV infection, we generated target cells with viral loads similar to those in spindle cells extracted from KS lesions. We show that latently KSHV-infected fibroblasts had normal levels of MHC-class I, ICAM-1, HLA-E and NKG2D ligand expression, were resistant to NK-cell natural cytotoxicity and were highly susceptible to killing by cytokine-activated immunocompetent NK cells. KSHV-infected fibroblasts expressed normal levels of IFN-γR1 and responded to exogenous IFN-γ by upregulating MHC class I, ICAM-1 and HLA-E and resisting activated NK-cell killing. These data demonstrate that physiologically relevant levels of latent KSHV infection in primary cells cause limited activation of resting NK cells and confer little specific resistance to control by activated NK cells.

## Introduction

Kaposi's sarcoma-associated herpesvirus (KSHV) infection is etiologically linked with the endothelial cell cancer Kaposi's sarcoma (KS), and with the rare B-cell lymphoproliferative disorders primary effusion lymphoma (PEL) and multicentric Castleman's disease (MCD) 1–3. KSHV exists in either a lytic or latent state facilitating life-long infection of the host 4. Individuals with KSHV infection have cellular and humoral immune responses to latent and lytic KSHV antigens 5–8.

KSHV infection is predominantly latent within KS lesions with the expression of a small number of genes by proliferating spindle cells of lymphatic endothelial cell (LEC) phenotype 9. While otherwise healthy KSHV-infected individuals may remain asymptomatic, the risk of developing KSHV-associated diseases is increased 20 000-fold in individuals who are immunosuppressed by HIV-1 infection or by 500-fold in patients receiving immunosuppressive drug therapy following allograft transplantation 10. AIDS-KS is aggressive and can involve the skin, the lymph nodes and the mucosa of the lungs and gut 3. Disease pathogenesis in AIDS-KS is linked to depletion of CD4^+^ T cells and the loss of KSHV-specific CD8^+^ cytotoxic T lymphocyte (CTL) responses 8, and treatment of AIDS-KS patients with highly active antiretroviral therapy (HAART) frequently induces KS remission 11 in association with recovery of anti-KSHV CTL responses 6.

Many viruses express factors (HIV-1 Nef 12, CMV-US-2, US-3, US-6, US-11 13, 14, EBV–EBNA1 15) which restrict viral clearance by interfering with MHC class I expression and viral peptide presentation to CD8^+^ CTLs. Early during lytic reactivation, KSHV avoids recognition by the expression of K3 and K5 ubiquitin ligases 16, 17. Both of these downregulate surface expression of HLA-A and HLA-B but K3 also downregulates the NK-cell inhibitory ligands HLA-C and HLA-E 16. Reduced expression of these HLA allotypes leaves the infected host cell vulnerable to lysis by NK cells which, unlike CD8^+^ CTLs, are primed to kill virally infected cells without requiring prior antigenic sensitisation 18. To avoid such killing by NK cells, KSHV uses K5 to reduce surface expression of ICAM-1 adhesion molecules and the NK-cell activatory ligand MHC class I-related chain A (MICA) 19. Furthermore, K5 and K3 block the effect of antiviral IFN-γ by downregulating the IFN-γR1 20. Thus, during lytic reactivation, KSHV effectively disables construction of the immunological synapse between a virally infected host cell and effector CTLs and NK cells. This effect has also been reported in early de novo latent KSHV infection as an outcome of concurrent lytic and latent gene expression 21, 22.

The importance of NK-cell immunity is demonstrated in the rare incidences of naturally NK-cell-deficient individuals who are highly vulnerable to herpesvirus infections whilst retaining immunity to bacterial and parasitic infections, 23. NK cells from AIDS-KS patients failing HAART and with active KS have low cytotoxicity against KSHV-infected body cavity B-cell lymphoma cell (BCBL-1) targets, whereas NK cells from healthy individuals, and from AIDS patients receiving HAART and with regressing KS, have high cytotoxicity, suggesting that effective control of KSHV infection in KS is linked with intact NK-cell function 24.

The extent to which KSHV-induced phenotypic changes affect the efficiency of NK-cell recognition and kill in the context of overall KSHV infection is unclear since previous studies have evaluated KSHV infection and NK-cell-mediated cytotoxicity using transfectants expressing only KSHV-K5 or K3 proteins 25, 26, or have used transformed KSHV-infected BCBL cell lines as target cells with no equivalent uninfected cells used as negative controls 24. To address this issue, we generated new target cells using primary dermal fibroblasts chronically infected with KSHV or uninfected. Using this system, we first determined whether chronic latent KSHV infection of primary, untransformed cells affects surface expression of MHC class I, ICAM-1, HLA-E and NKG2D ligands involved in NK-cell target recognition and activation. Secondly, we investigated whether such changes in phenotype enabled resistance to killing by NK cells. Thirdly, since NK cells control virus replication through anti-viral cytokine secretion, we investigated the effect of chronic latent KSHV infection on responsiveness to IFN-γ.

## Results

### Latent infection of dermal neonatal fibroblasts with KSHV

**Figure 1:**
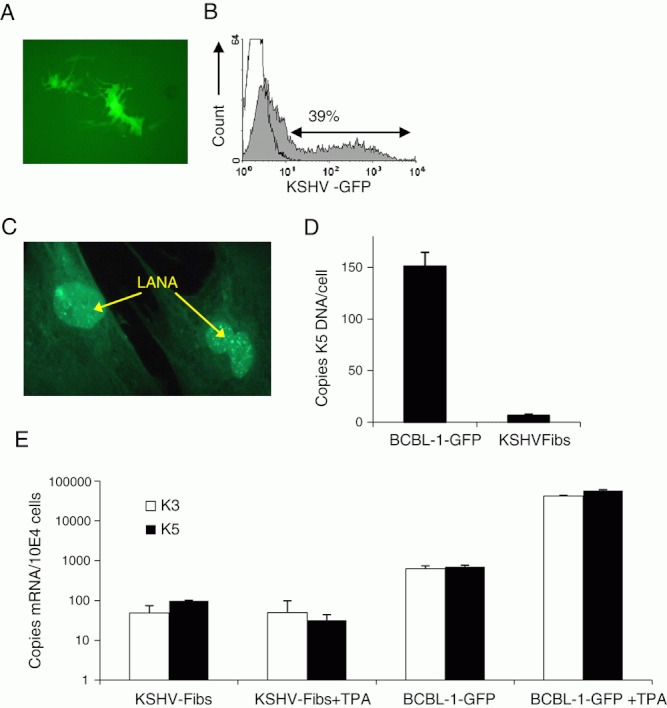
Latently infected primary neonatal dermal KSHV-GFP fibroblast cells generated through co-culture with BCBL-1-GFP cells. (A) Micrograph of a single KSHV-GFP infected fibroblast 24 h after co-culture with BCBL-1-GFP cells (photographed at 200× magnification under phase-contrast and UV fluorescence). (B) Flow cytometric analysis of GFP expression by KSHV-GFP-infected fibroblasts (grey histogram) after G418 selection relative to uninfected fibroblasts (open histogram). (C) IFA detection of ORF 73 LANA protein in nuclei of KSHV-infected fibroblasts (photographed at 200× magnification under phase-contrast and UV fluorescence). (A–C) are representative of five independent experiments. (D) Total cellular KSHV viral load determined by quantitative real-time PCR analysis of K5 DNA by chronically KSHV-infected fibroblasts and BCBL-1 cells. Data are shown as mean copies/cell±SD (*n*=3). (E) Expression of KSHV K3 and K5 lytic cycle associated mRNA production in resting and TPA-stimulated KSHV-infected fibroblasts and BCBL-1-GFP cells determined by quantitative real-time RT-PCR (qRT-PCR). KSHV-infected fibroblasts or BCBL-1-GFP cells were stimulated with TPA before harvesting for mRNA analysis by qRT-PCR. Data shown are the mean±SD of copies of mRNA/10^4^ cells upon correction for cell input by qRT-PCR for GAPDH and are summarised from four experiments.

To study the susceptibility of primary cells latently infected with KSHV to NK-cell lysis, it was necessary to generate sufficient identifiably infected cells while at the same time maintaining viral latency. We made use of a system employing co-culture of *O*-tetradecanoylphorbol 13 acetate (TPA)-activated KSHV-positive EBV-negative BCBL-1 cells in order to infect primary cell lines whilst avoiding the potential induction of the lytic cycle by residual TPA associated with large-scale preparations of KSHV 27. Furthermore, infected primary cells were readily detected and expanded by the use of recombinant GFP-tagged KSHV and selection using G418 as originally used to select the BCBL-1-GFP cells 28. In initial studies, primary dermal vascular endothelial cells were successfully infected with KSHV-GFP (data not shown). However, subsequent expansion of these cells failed under selection with G418, and insufficient numbers of identifiable infected cells were obtained to perform NK-cell assays. We, therefore, used primary dermal fibroblasts as targets as these can be effectively infected and expanded and are of a similar size to primary endothelial cells with similar levels of expression of MHC class I and ICAM-1. Overnight co-culture at a BCBL-1-GFP:fibroblast ratio of 10:1 induced KSHV-GFP expression by <1% of plated fibroblasts (Fig. 1A). After 14–21 days of G418 selection, the proportion of KSHV-GFP^+^ fibroblasts had increased to 25–40% with a wide (2 logs) range of GFP expression (Fig. 1B) and identical morphology to uninfected fibroblasts.

Expanded KSHV-infected fibroblasts were confirmed to be latently infected by punctuate nuclear latent nuclear antigen (LANA) staining (Fig. 1C). After G418 selection, LANA was detected in all cells indicating that both GFP^–^ and GFP^+^ fibroblasts had been infected with recombinant virus. In contrast, virtually all G418-treated uninfected fibroblasts were killed within the 2- to 3-wk G418 selection period (data not shown). KSHV viral load was measured by quantitative real-time PCR for K5 DNA (Fig. 1D). The mean viral load for KSHV-infected fibroblasts was 6.5±1.2 copies/cell (*n*=3) compared with 151±14 copies/cell (*n*=3) for the BCBL-1-GFP cells used to infect the fibroblasts. Analysis of viral mRNA by quantitative RT-PCR showed that latently infected KSHV-infected fibroblasts had very low levels of constitutive production of mRNA encoding early lytic cycle associated genes K3 or K5 (49±25 and 59±2 copies/10^4^ cells (*n*=4), respectively). This was <10% of the basal levels in BCBL-1-GFP cells (630±111 and 704±65 copies/10^4^ cells of K3 and K5, respectively). Stimulation of KSHV-infected fibroblasts with TPA failed to induce K3 and K5 mRNA production in contrast to BCBL-1-GFP cells where TPA induced a 60- and 80-fold increase in K3 and K5 mRNA production, respectively (Fig. 1E). These data suggest that KSHV-infected fibroblasts were predominantly latently infected with very low levels of spontaneous lytic cycle activity compared with BCBL-1-GFP cells.

### Phenotype of KSHV-infected fibroblasts

**Figure 2:**
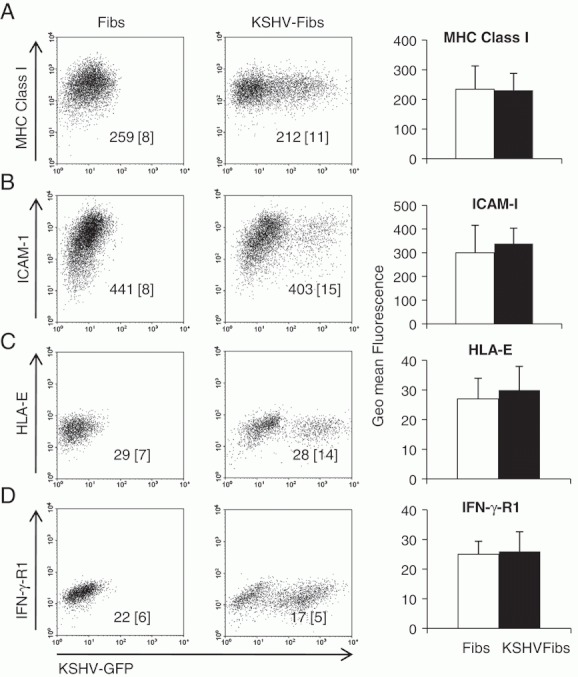
Phenotype of latently infected KSHV-GFP fibroblasts. Expression of (A) MHC class I, (B) ICAM-1, (C) HLA-E and (D) IFN-γR1 in latently KSHV infected and passage-matched uninfected control fibroblasts. Data shown are representative of seven (for MHC class I and ICAM-1), four and five experiments, respectively. Values inserted in the plots are *y*-geo-mean fluorescence. The values in square brackets [ ] are the *y*-geo-mean fluorescence for the respective isotype control. The histograms on the right-hand side of each representative plot show summarised data for control and KSHV-infected fibroblasts in terms of mean±SD geomean fluorescence for MHC class I (*n*=7), ICAM-1 (*n*=7), HLA-E (*n*=4) and IFN-γ-R1 (*n*=5).

De novo KSHV infection has been reported to cause rapid downregulation of MHC class I, ICAM-1 and IFN-γR1 which is thought to protect KSHV-infected cells from killing by NK cells and cytotoxic T cells 20, 29. Figures 2A and 2B show that chronically KSHV-infected fibroblasts and uninfected control fibroblasts had similar high geo-mean fluorescence levels of MHC class I (231±57 and 234±79, respectively) and ICAM-1 (340±65 and 300±116, respectively). Following the same pattern, KSHV-infected fibroblasts and control uninfected fibroblasts had similar low levels of expression of the non-classical MHC class I molecule HLA-E (Fig. 2C and D, 30±8 and 27±7, respectively) which inhibits NK-cell activation, and IFN-γR1 (geo-mean fluorescence levels of 26±7 and 25±4, respectively). These data suggest that chronic KSHV infection of fibroblasts has no sustained effect on the expression of MHC class I, ICAM-1, HLA-E or IFN-γR1.

### Latently KSHV-infected cells are susceptible to killing by activated NK cells

**Figure 3:**
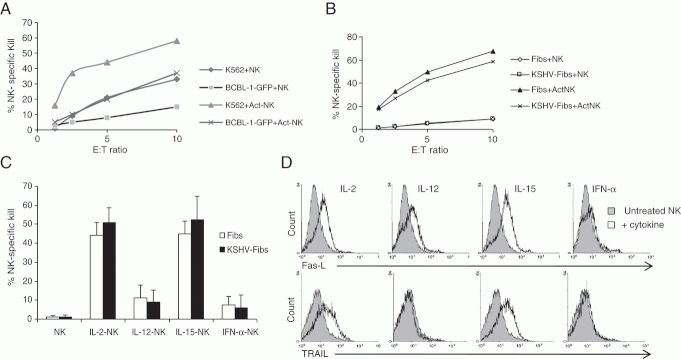
Lysis of latently infected BCBL-1-GFP cells and KSHV-GFP fibroblasts by activated NK cells. (A) Susceptibility of BCBL-1-GFP cells to lysis by NK cells. Lysis of BCBL-1-GFP cells by resting (NK) or IL-15-activated allogeneic NK cells was determined after 4 h of culture. K562 cells were used as a positive control. The data shown are the mean of duplicate determinations and are representative of four independent experiments. (B) Susceptibility of latently KSHV-infected fibroblasts to natural and IL-15-activated NK cell-mediated killing. KSHV-infected fibroblasts and uninfected fibroblasts were co-cultured with resting or activated NK cells for 18 h prior to determination of cytotoxicity. The data shown are the mean of duplicate determinations of NK-specific kill and are representative of four independent experiments. (C) Analysis of lysis of KSHV-infected fibroblasts by NK cells activated with IL-2, IL-12, IL-15 or IFN-α. Data shown are the mean+SD of NK-specific kill of targets (E:T ratio of 5:1) from four separate experiments. (D) Flow cytometric analysis of Fas-L (upper panel) and TRAIL (lower panel) expression by untreated (filled grey histogram) and cytokine-treated NK cells (open histogram). NK cells were cultured with or without the cytokines indicated for 24 h. The data shown are representative of two analyses.

BCBL-1 cells have been demonstrated to be susceptible to NK-cell lysis 24. We therefore investigated the susceptibility of BCBL-1 cells infected with KSHV-GFP to NK-cell lysis. Only weak natural killing of BCBL-1GFP cells was observed compared with NK-sensitive K562 cells (Fig. 3A). Activation of NK cells with IL-15 resulted in increased lysis of both BCBL-1-GFP cells and K562. Figure 3B shows that both uninfected and KSHV-infected allogeneic fibroblasts had low levels of susceptibility to natural killing by resting NK cells even after 20 h culture. Furthermore, KSHV-infected fibroblasts had similar high levels of susceptibility to IL-15-activated NK cells, indicating that latent infection did not confer significant resistance to kill. In addition to IL-15, NK cells are stimulated by IL-2 from activated T cells 30 and by IL-12 and IFN-α from activated macrophages and dendritic cells 31. Figure 3C shows that NK cells activated by IL-15 and IL-2 were three-fold more potent killers of control and KSHV-infected fibroblasts than NK cells activated with IL-12 or IFN-α. Since human neonatal dermal fibroblasts express the apoptosis receptors Fas 32 and TRAIL-receptor-2 33, we examined the expression of their respective ligands on NK cells treated with or without cytokines. Flow cytometric analysis revealed that the superior cytotoxicity of IL-2- and IL-15-activated NK cells against fibroblast targets was linked with increased NK-cell expression of Fas ligand and TRAIL ligand (Fig. 3D). However, this probably reflected the overall enhanced cytolytic capability of IL-2- and IL-15-activated NK cells since antibody blockade of Fas-L and TRAIL alone and in combination with anti-LFA-1 had no significant protective effect (Supporting Information Fig. 1). That upregulation of Fas-L and TRAIL alone was insufficient for killing of resting uninfected and latently KSHV-infected fibroblast targets suggests that other mechanisms of cytolysis are involved 34. Thus, chronic latent KSHV infection of fibroblasts confers no specific protection against cytotoxic effects of activated NK cells.

### KSHV-infected fibroblasts express normal levels of ligands for NKG2D

**Figure 4:**
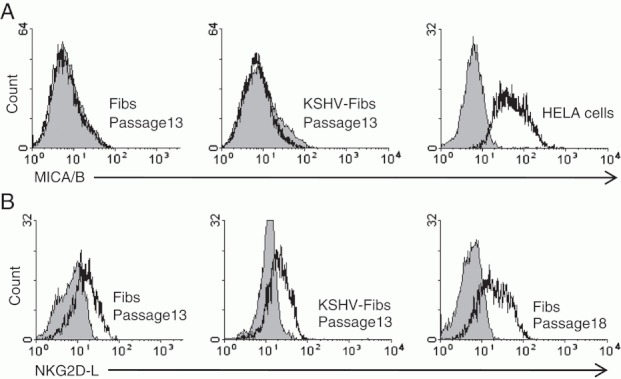
Expression of NKG2D ligands by KSHV-infected fibroblasts. (A) Lack of surface expression of MICA/B expression (open histogram) by control uninfected and KSHV-infected fibroblasts compared with HeLa cells. The data shown are representative of three experiments. (B) Expression of NKG2D ligands (open histogram) by near senescent uninfected fibroblasts compared with passage-matched control uninfected and KSHV-infected fibroblasts. The data shown are representative of three experiments.

NK cells recognise and clear damaged cells through activation of the NKG2D receptor by stress-induced ligands including the non-classical MHC class I chain-related molecule A and B (MICA/B) and the ULBP family. Recent data suggest that latently KSHV-infected BCBL-1 cells are partially protected from NK cells by downregulation of MICB through a KSHV encoded microRNA-mediated mechanism 35. We therefore examined the expression of MICA/B on control uninfected and KSHV-infected fibroblasts by flow cytometry. Although high levels of expression of MICA/B were found on HeLa cell positive controls, we could not detect MICA/B expression on fibroblasts with or without KSHV infection (Fig. 4A).

In an alternative approach, we used NKG2D-Fc as a probe to detect surface expression of all NKG2D ligands. There was an increased expression of NKG2D ligands on near-senescent passage 18 fibroblasts compared with passage 13 fibroblasts, but no detectable effect of chronic latent KSHV infection on NKG2D ligand expression (Fig. 4B).

IFN-γ-induced protection of KSHV-infected fibroblasts from kill by NK cells is associated with downregulated ORF50 mRNA and upregulated MHC class I, ICAM-1 and HLA-E expression.

**Figure 5:**
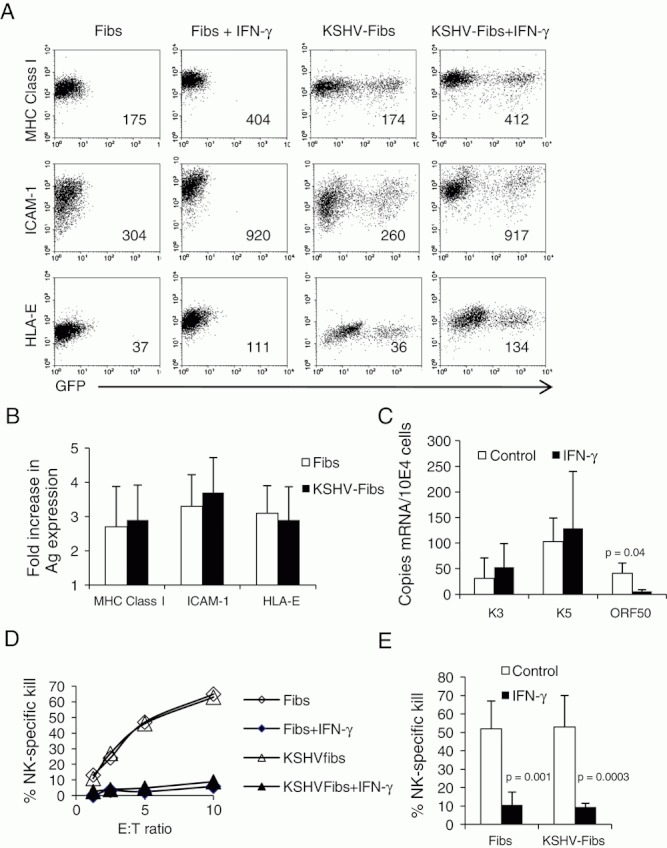
IFN-γ-induced upregulation of MHC class I, ICAM-1 and HLA-E and resistance to cell killing. (A) Effect of 48 h of treatment with IFN-γ (10 ng/mL) on expression of MHC class I, ICAM-1 and HLA-E by control uninfected and KSHV-infected fibroblasts. Values inserted in the plots are *y*-geo-mean fluorescence values and are representative of four independent experiments. (B) Summarised data show fold increase in IFN-γ-induced upregulation of antigen by control and KSHV-infected fibroblasts. Data presented as mean±SD (*n*=4). (C) Effect of IFN-γ on baseline expression of ORF50 but not KSHV K3, K5 lytic cycle associated mRNA production. KSHV-infected fibroblasts were stimulated with IFN-γ for 48 h before harvesting for mRNA analysis by qRT-PCR. Data shown are the mean±SD (*n*=4) of copies of mRNA/10^4^ cells upon correction for cell input by qRT-PCR for GAPDH. (D) Effect of IFN-γ treatment on killing by IL-15-activated NK cells. KSHV-infected fibroblasts and uninfected fibroblasts were treated with or without IFN-γ for 48 h prior to co-culture with activated NK cells for 18 h. The data shown are the mean of duplicate determinations of NK-specific kill and are representative of four independent experiments. (E) Summarised data show the mean±SD NK-specific kill of control and IFN-γ-treated targets (E:T ratio of 5:1) from three separate experiments. Statistical significance was determined by Student's *t*-test.

Apart from direct lysis of virally infected cells, activated NK cells can control levels of virus production by producing anti-viral IFN-γ, which also strongly upregulates MHC class I and ICAM-1 expression. However, IFN-γ has also been reported to induce KSHV lytic cycle replication 36, which downregulates these molecules. Furthermore, IFN-γ sensitises virally infected cells to TRAIL-induced apoptosis 33. We, therefore, investigated the effect of IFN-γ treatment on cell phenotype, KSHV lytic cycle mRNA and susceptibility of KSHV-infected cells to killing by activated NK cells. IFN-γ induced a rapid change in both KSHV-infected and control uninfected fibroblasts to a spindle cell type morphology (data not shown) and a marked three-fold upregulation of MHC class I, ICAM-1 and HLA-E (Fig. 5A and B). Instead of KSHV lytic cycle induction, IFN-γ significantly reduced ORF50 (the master regulator of KSHV lytic reactivation from latency) mRNA levels (*p*=0.04), but not K3 or K5 mRNA (Fig. 5C). IFN-γ pre-treatment strongly inhibited killing of both KSHV-infected and control uninfected cells by IL-15-activated NK cells (Fig. 5D and E; *p*=0.0003 and 0.001, respectively), These data suggest that in addition to IFN-γR1 expression, latently KSHV-infected fibroblasts have intact or normal levels of IFN-γ responsiveness, which modulates the expression of surface antigens crucial for mediating NK-cell activation, KSHV viral lytic gene expression and susceptibility to killing by activated NK cells.

## Discussion

Persistent KSHV infection of endothelial cells within KS lesions is predominantly latent with low levels of lytic activity 37. The involvement of NK cells in controlling KS is suggested by observations that patients with active KS have poor cytotoxicity against K562 and KSHV-infected BCBL cell targets, and that regression of KS in AIDS-KS patients responding to HAART is linked to recovery of NK-cell cytotoxicity 24. The ideal target cell for investigating how susceptible KSHV-infected cells are to NK-cell control would be spindle cells derived from KS lesions, but there are two main barriers to this. First, KSHV-infected endothelial cells are not transformed and KSHV genomes are rapidly lost after a few passages in vitro leading to an outgrowth of KSHV^–^ cells 38. Second, confirmation of KSHV infection of KS spindles is difficult to confirm without rendering the cell unviable for cytotoxicity studies.

In an alternative approach, we have established an in vitro model system for studying the ability of NK cells from immunocompetent individuals to kill primary human fibroblasts which have been chronically latently infected with KSHV. We found that resting NK cells exhibited only weak natural cytotoxicity against KSHV-infected dermal fibroblasts with no additional killing being observed over that seen when uninfected fibroblasts were used as targets. Previous studies have demonstrated NK-cell killing of KSHV-infected BCBL-1 PEL cells, an observation confirmed in the current study 24. However, in the case of such tumours it was not possible to distinguish any potential modulating effects of latent KSHV infection on the NK-cell response to the cell line, due to the lack of uninfected control cells. Observations from our model suggest that chronic latent infection of primary cells with KSHV does not induce additional natural kill activity or prevent cytotoxicity by cytokine-activated NK cells.

Three weeks after de novo infection, we found that surface expression of ICAM-1, MHC-class I, HLA-E, IFN-γR1 and NKG2D-ligands by both GFP^–^ and GFP^+^ KSHV-infected cells in established persistent latency was the same as in uninfected controls, consistent with a lack of modulation of NK-cell cytotoxicity. Thus, chronic latent KSHV infection of fibroblasts by co-culture had no detectable sustained effect on basal surface expression of antigens involved in immunological synapse formation or ligands for NK-cell activation and inhibition. This may reflect the situation in vivo where KSHV infection is asymptomatic. In contrast, gene expression microarray data derived from the nodules of AIDS-KS patients suggest that relative mRNA expression for the activating NKG2D ligands MICB and ULBP2 is significantly increased compared with healthy skin 39. Our data would suggest that this is not due to the direct effects of latent KSHV infection, but perhaps through the advanced KS lesion being an inflamed hypoxic environment 40.

The lack of modulation of MHC class I, ICAM-1, NKG2D ligands, IFN-γR1 and HLA-E in our primary fibroblast system is likely due to the relatively low initial KSHV viral load obtained through co-culture compared with the use of highly concentrated KSHV viral supernatant, and because of the dilution of KSHV episomes among infected daughter cells. The advantages of the co-culture method are the reported high efficiency of cell–cell transfer of virus (31), and most importantly its low cytopathic effect as viable target cells are essential for sensitive NK-cell cytotoxicity assays. Recent studies using highly sensitive multispectral imaging flow cytometry confirm that downregulation of MHC class I and ICAM-1 following de novo KSHV infection correlates with intracellular viral load 22. It is of note that the viral load of 6 copies/cell in KSHV-infected fibroblasts reported here is of a similar magnitude to an estimate of 3.2 copies/cell in KS lesions (43). In comparison, the BCBL-1-GFP cells used to initially infect the fibroblasts had a mean viral load of 150 copies/cell and tenfold greater production of lytic K3 and K5 mRNA. Accordingly, latently infected BCBL-1-GFP cells had low levels of ICAM-1 and MHC class I expression (data not shown) and were more resistant than latently infected KSHV-Fibs to activated NK-cell killing, possibly mediated in part through the recently described KSHV microRNA miR-K12-7 targeting of MICB mRNA 35.

Our data suggest that low levels of gene expression in chronic latent KSHV infection of fibroblasts appear to confer little protection from activated NK-cell killing. However, during de novo KSHV infection there is concurrent expression of high levels of latent genes and production of a selected range of lytic factors with anti-apoptotic and immune modulating activity, but not lytic genes coding for viral DNA synthesis or viral structural proteins (28). Downregulation of MHC class I is critical because high levels of production of viral antigens during this period would render the newly infected cell highly vulnerable to kill by CTLs. Thereafter, expression of lytic genes is progressively reduced until only latent gene expression persists. However, early lytic factor K5 protein can be detected up to 5 days after initial infection potentially helping the infected host cell to escape kill by NK cells and CTLs. Indeed, KSHV-induced ICAM-1 and MHC class I downregulation have been reported to persist for up to 23 days in primary dermal microvascular endothelial cells 29. In contrast, in latently infected primary fibroblasts, MHC class I, ICAM-1, NK-cell activatory ligands and IFN-γR1 may have been downregulated during early infection, and normalisation of expression of these molecules may occur under conditions of low KSHV viral copy number and low levels of KSHV gene expression or possibly through the upregulatory effect on MHC class I expression by KSHV v-FLIP 39.

Although microvascular dermal endothelial cells, the natural initial target of KSHV infection in KS, exhibit some phenotypic and functional differences from dermal fibroblasts, such cells may be similarly protected from cytolysis under conditions of low copy number latent infection. Further, where NK cells are activated by cells undergoing low levels of KSHV reactivation, our data demonstrating normal responsiveness of latently KSHV-infected cells to IFN-γ, suggests that neighbouring cells, latently infected with KSHV, although inhibited from activating lytic viral replication, may be protected from NK-cell killing. This might be through a bystander upregulation of HLA-E, which ligates the inhibitory NKG2A–CD94 complex combined with upregulation of HLA-C which ligates the inhibitory NK-cell receptor CD158a/b. The role of HLA-E and HLA-C in IFN-γ-induced resistance to NK-cell killing merits further investigation as on the other hand, NK cells from patients with chronic KS and in healthy HCMV seropositive individuals have increased numbers of NK cells expressing the activatory NKG2C–CD94 complex, which would be predicted to kill HLA-E expressing targets 41, 42.

The potent cytolytic effect of activated NK cells from healthy individuals on primary cells with low levels of chronic latent KSHV infection may partially explain why, in immunocompetent individuals, KSHV infection is essentially asymptomatic and with very low viral load. In this respect, the impact of NK cells from immunosuppressed individuals on primary cells with low levels of KSHV viral load is of immediate interest. Cytokine-mediated activation of NK cells by IL-15 in such immunosuppressed patients with persistent KS, particularly during HIV-1 infection, may be of immunotherapeutic value 43.

## Materials and methods

### Cells and culture

Primary human neonatal dermal fibroblasts were obtained from Lonza (Wokingham, UK) and cultured in FGM-2 bulletkit medium (Lonza). The source of KSHV (GFP-KSHV) was the GFP-recombinant KSHV^+^, EBV^–^, BCBL-1 cell line KSHV152-BCBL-1 28; a gift from Dr Jeff Vieira, University of Washington, Seattle, USA. The GFP-BCBL-1 cells used had been cloned for high GFP expression and were cultured in RPMI 1640 medium (Invitrogen, Paisley, UK) supplemented with 10% FCS (Sigma, Portsmouth, UK) 2 mM L-glutamine and penicillin/streptomycin (250 μg/mL) at a density of from 5×10^5^ to 1×10^6^ cells/mL.

### KSHV infection of fibroblasts

Passage 10 fibroblasts were cultured to 90% confluence in 24-well plates (Costar), and co-cultured with or without washed TPA (20 ng/mL; Sigma-Aldrich, Poole, Dorset, UK) stimulated GFP-BCBL-1 cells 27 at a ratio of 10 GFP-BCBL-1 cells:1 fibroblast. After 18 h co-culture, GFP-BCBL-1 cells were removed by washing with PBD and fibroblasts recultured in FGM-2. After 4 days, G418 (250 μg/mL; Sigma) was added to select out GFP-KSHV-infected fibroblasts, which were expanded thereafter in FGM-2 bulletkit medium in the presence of G418. KSHV infection was confirmed by the detection of ORF 73/ LANA protein by IFA as previously described 44. Cells were examined under phase contrast using UV fluorescent microscopy. Aliquots of 2×10^5^ cells were sub-cultured in the absence of G418 for 5 days before all experiments.

### Flow cytometric analysis

MHC class I, ICAM-1, MICA/B, HLA-E, IFN-γR1 and NKG2D ligand expression by trypsinised uninfected and KSHV-infected fibroblasts was assessed by flow cytometry. About, 3×10^4^ cells were harvested and stained directly with anti-HLA-ABC or ICAM-1, conjugated to PE or APC (Pharmingen, Oxford, UK) or to MICA/B conjugated to APC (R&D Systems, Oxford, UK). Indirect staining for HLA-E was performed using unconjugated anti-HLA-E (clone 3D12, a gift from Dr Dan Geraghty, Fred Hutchinson Cancer Research Centre, Seattle, WA, USA). Indirect staining for IFN-γR1 expression was performed using unconjugated anti-IFN-γR1 (Santa Cruz Biotechnology, CA, USA). HLA-E and IFN-γR1 antigens were detected with biotin-conjugated anti-mouse IgG1 and streptavidin-APC (BD Pharmingen). Indirect staining for NKG2D ligand expression was performed using NKG2D-Fc (R&D Systems) and detected using biotin-conjugated anti-human IgG1 and streptavidin-APC (BD Pharmingen). Indirect staining for Fas-L and TRAIL expression by NK cells was performed using antibodies from R&D Systems and detected using biotin-conjugated anti-mouse IgG1 and streptavidin-APC.

Flow cytometry was performed with a Becton Dickinson FACScalibur cytometer using standard CellQuestPro acquisition software (Becton Dickinson, Mountain View, CA, USA).

### Preparation of NK effector cells

NK effector cells were purified by negative selection using the Miltenyi Biotec NK-cell isolation kit (Bergisch Gladbach, Germany) from Ficoll Hypaque-separated PBMCs donated by healthy adult volunteers (according to local ethical committee approval). NK cells (purity>95% CD3^–^ and CD56^+^) were cultured overnight at a density of 10^6^/mL in 24-well plates in complete medium (RPMI 1640 medium (Invitrogen) supplemented with 10% human AB serum, 2 mM L-glutamine) with or without the NK-cell activating monokines: IL-12, IL-15 (20 ng/mL), IFN-α (1000 IU) or with IL-2 (20 ng/mL; R&D Systems).

### Colorimetric cytotoxicity assay

NK cells treated with or without cytokines were added at various effector:target (E/T) ratios to uninfected and KSHV-infected fibroblast target cells at a density of 1×10^4^ cells per well and cultured in complete medium in flat-bottomed 96-well plates. The plates were spun at 100×*g* for 3 min to enhance effector–target cell contact. After 18 h of culture, the plates were re-spun, and lactate dehydrogenase (LDH) release in harvested supernatants was measured using the Cytotox 96′ kit (Promega, USA) according to the manufacturer's instructions. In experiments using non-adherent PEL cells and K562 cells as targets, assays were conducted in round-bottomed 96-well plates and cultured for 4 h before harvesting for LDH release.

### Quantitative real-time PCR for KSHV viral load and lytic K3 and K5 mRNA

KSHV viral load and viral mRNA production were assessed by real-time quantitative PCR (Q-PCR) using self-probing ‘scorpion’ primers 45. Total RNA was extracted from cells using the Qiagen RNAeasy kit (Crawley, Sussex, UK) and subjected to two rounds of DNAase digestion and reverse transcribed into cDNA using a Prostar first strand generation kit (Stratagene, USA). Aliquots of 2 μL of cDNA template were subjected to Q-PCR using FAM-labelled scorpion primers (ATDBio, University of Southampton, UK). Genomic DNA was extracted using the Qiagen DNeasy tissue kit and subjected to Q-PCR.

The scorpion primers used were (where f is the FAM fluorophore, que is the quencher and heg is the blocker):

GAPDH: forward: 5′-f-CCGCGGAGGACTCATGACCACAGCCGCGG-que-heg-GGGGCCATCCACAGT CTTCT-3′; reverse; 5′-GCCTCCTGCACCACCAACTG-3′

K3: forward: 5′-f-CCCTGTGCATCCACAGGG-que-heg-GGAGCTCGGAAATGAGAGATTTAGA-3′; reverse: 5′-GAGCCAGGTGCTTAAACAAC-3′;

K5: forward: 5′-f-TCGCGGTACAGGCGCGA-que-heg-GTGGGGAACGAGGGCATACA-3′; reverse: 5′-GTTAGCCAAGTGCTTAAACACT-3′;

ORF50: forward: 5′-f-CCCGGTGGTAATTGGCCGGG-que-heg-CATCACCGGTTCTGCTGAGA-3′; reverse: 5′-TACCATGGAAGCCGGCAACA-3′.

PCR conditions were as follows: a series of 46-cycle two-step PCRs (15 s denaturation and 20 s annealing) was carried out using a Lightcycler (Roche, USA). The final PCR reaction mixture consisted of 2 μL cDNA template, 0.5 U of *Taq* polymerase (Promega), 4 μL of dNTPs (4 mM; Stratagene), 0.4 μL of scorpion sense and anti-sense primers (5 μM each), 2 μL of reaction buffer and 2 μL of 25 mM MgCl_2_ (Promega). The final volume of 20 μL was made up with nuclease-free H_2_O.The annealing temperatures for GAPDH, K3 and K5 were: 56, 60, 60 and 58°C, respectively.

Target template generation: GAPDH and viral mRNA levels were expressed as the number of copies detected in each sample. This was calculated using standard curves from serial dilutions of target template. Target template was purified PCR product generated using regular non-scorpion-conjugated versions of the forward primer. PCR products were purified using the QIAquick PCR purification kit (Crawley, UK) according to the manufacturer's instructions. Threshold cycle values were converted to copies of template by reference to a standard curve created by the LightCycler software. GAPDH was used as a reference gene to correct for template input.

### Statistical analysis

Comparisons between groups were made using the two-tailed Student's *t*-test. A value of *p*<0.05 was considered significant.

## Abbreviations

BCBL-1: body cavity B-cell lymphoma cell
HAART: highly active antiretroviral therapy
KS: Kaposi' sarcoma
KSHV: Kaposi's sarcoma-associated herpesvirus
LANA: latent nuclear antigen
MICA: MHC class I-related chain A
MICB: MHC class Irelated chain B
PEL: primary effusion lymphoma
TPA: Otetradecanoylphorbol 13 acetate
