# Supplementary material for: Killing of Kaposi's sarcoma-associated herpesvirus-infected fibroblasts during latent infection by activated natural killer cells
Source: Eur J Immunol. 2011 May 27;41(7):1958–68. doi: 10.1002/eji.201040661 (PMC3485667; doi:10.1002/eji.201040661)
Supplement: Supplementary file 1 [file eji0041-1958-SD1.pdf]

European Journal of Immunology

Supporting Information

for

DOI 10.1002/eji.201040661

Killing of Kaposi's sarcoma-associated herpesvirus-infected fibroblasts during latent infection by activated natural killer cells

Nick C. Matthews, Martin R. Goodier, Rebecca C. Robey, Mark Bower and Frances M. Gotch

© 2011 WILEY-VCH Verlag GmbH & Co. KGaA, Weinheim

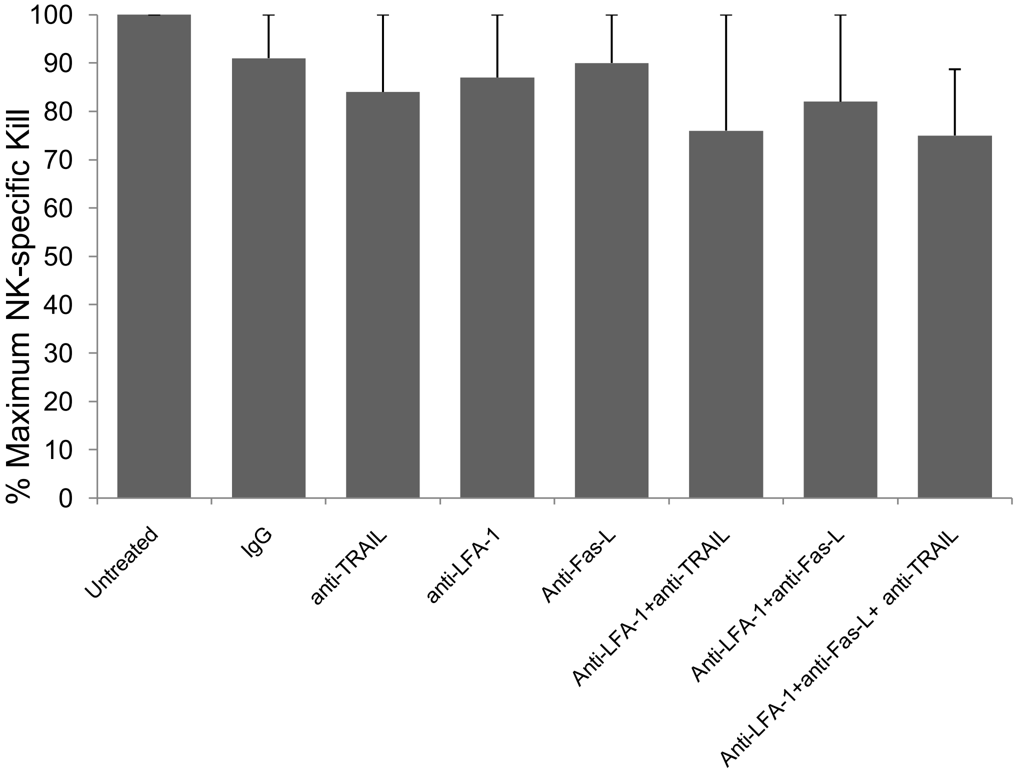

Supplemental figure 1

Lack of inhibitory effect of anti-TRAIL, anti-LFA-1 and anti-Fas on killing of KSHV-infected fibroblasts by NK cells after 18h culture. IL-15 activated NK cells were pretreated with antibodies for 1h before culture with target cells Data shown are the normalised mean +/- SD of NK specific kill of targets (E:T ratio of 5:1) summarised from 4 separate experiments.
